# Preparation of Mesoporous Mn–Ce–Ti–O Aerogels by a One-Pot Sol–Gel Method for Selective Catalytic Reduction of NO with NH_3_

**DOI:** 10.3390/ma13020475

**Published:** 2020-01-19

**Authors:** Yabin Wei, Shuangling Jin, Rui Zhang, Weifeng Li, Jiangcan Wang, Shuo Yang, He Wang, Minghe Yang, Yan Liu, Wenming Qiao, Licheng Ling, Minglin Jin

**Affiliations:** 1 School of Materials Science and Engineering, Shanghai Institute of Technology, Shanghai 201418, China; 176081103@mail.sit.edu.cn (Y.W.); 176081107@mail.sit.edu.cn (W.L.); 176082112@mail.sit.edu.cn (J.W.); 176082108@mail.sit.edu.cn (S.Y.); 186081133@mail.sit.edu.cn (H.W.); 186081138@mail.sit.edu.cn (M.Y.); lyan@sit.edu.cn (Y.L.); jml@sit.edu.cn (M.J.); 2 State Key Laboratory of Chemical Engineering, East China University of Science and Technology, Shanghai 200237, China; qiaowm@ecust.edu.cn (W.Q.); lchling@ecust.edu.cn (L.L.)

**Keywords:** Mn–Ce–Ti–O composite aerogel, mesopore size, NH_3_–SCR, apparent activation energy, SO_2_ resistance

## Abstract

Novel Mn–Ce–Ti–O composite aerogels with large mesopore size were prepared via a one-pot sol–gel method by using propylene oxide as a network gel inducer and ethyl acetoacetate as a complexing agent. The effect of calcination temperature (400, 500, 600, and 700 °C) on the NH_3_–selective catalytic reduction (SCR) performance of the obtained Mn–Ce–Ti–O composite aerogels was investigated. The results show that the Mn–Ce–Ti–O catalyst calcined at 600 °C exhibits the highest NH_3_–SCR activity and lowest apparent activation energy due to its most abundant Lewis acid sites and best reducibility. The NO conversion of the MCTO-600 catalyst maintains 100% at 200 °C in the presence of 100 ppm SO_2_, showing the superior resistance to SO_2_ poisoning as compared with the MnO_x_–CeO_2_–TiO_2_ catalysts reported the literature. This should be mainly attributed to its large mesopore sizes with an average pore size of 32 nm and abundant Lewis acid sites. The former fact facilitates the decomposition of NH_4_HSO_4_, and the latter fact reduces vapor pressure of NH_3_. The NH_3_–SCR process on the MCTO-600 catalyst follows both the Eley–Rideal (E–R) mechanism and the Langmuir–Hinshelwood (L–H) mechanism.

## 1. Introduction

Nitrogen oxides (NO_x_), as major air pollutants from the combustion of fossil fuels, cause serious environmental problems, such as acid rain, photochemical smog, and ozone depletion [1,2]. At present, many denitration technologies have been developed to remove NO_x_ to meet stringent legislation requirements, such as selective catalytic reduction (SCR) [1,2,3,4,5,6], selective non-catalytic reduction (SNCR) [7], and absorption [8], among which the selective catalytic reduction with NH_3_ (NH_3_–SCR) is one of the most promising approaches for NO_x_ removal owing to its high denitration efficiency and relatively low cost. Vanadium–titanium catalysts are the most commonly used catalysts currently in industry to remove NO_x_ from stationary sources, including V_2_O_5_–WO_3_/TiO_2_ and V_2_O_5_–WO_3_(MoO_3_)/TiO_2_ [9,10,11,12]. However, to satisfy the working conditions of the V-based catalysts, the current SCR denitration device has to be installed upstream of the electrostatic precipitator and the desulfurization device to avoid the deactivation of catalysts by high-concentration fly ash, alkali metals, and SO_2_ [13], to increase the life time of the catalysts and to reduce the operating costs of denitration. In addition, the V-based catalysts have some inevitable disadvantages, such as the toxicity of VO_x_, a narrow operating temperature window (300–400 °C), and a low N_2_ selectivity [6,14,15]. Therefore, it is of great practical importance to develop catalysts with high catalytic activity and good SO_2_ resistance under low-temperature conditions (<200 °C).

The MnO_x_–based catalysts have been considered as promising candidates for NO removal by the NH_3_–SCR method owing to their inherent environmentally benign nature and superior catalytic activity [13,14,15,16,17,18,19,20,21]. As an additive, CeO_2_ can further enhance the catalytic activity of MnO_x_ in the low temperature range because of its unique oxygen storage capacity and redox performance [22,23]. In addition, CeO_2_ can enhance the SO_2_ tolerance of MnO_x_–based catalysts, because CeO_2_ can preferentially react with SO_2_ to reduce the sulfation of MnO_x_ at the expense of the active CeO_2_ component [24]. Extensive efforts have also been made to further improve the SO_2_ resistance of MnO_x_–CeO_2_ catalysts, such as inhibiting the SO_2_ adsorption/oxidation and enhancing the adsorption of active intermediate species by introducing functional promoters [25]. Due to the relatively weak interaction between TiO_2_ and SO_2_, TiO_2_ can act as a protective constituent to inhibit the adsorption of SO_2_; especially, the coating of a TiO_2_ shell on MnO_x_–CeO_2_ is demonstrated to be a good method to improve the SO_2_ resistance of the catalysts [1,26]. Moreover, modification with Co, Ni, Fe, and Zn can increase the formation of nitrate species and NO_2_ on the surface of the MnO_x_–CeO_2_ catalysts, thereby enhancing the SO_2_ resistance [4,13,27]. In addition, it is proved that the pore texture also plays an important role in SO_2_ resistance of the catalysts [28,29,30,31]. Soh et al. suggested that the increase of pore size and specific surface area for V_2_O_5_/Al_2_O_3_ catalysts can reduce the deactivation rate of active sites [29]. Similarly, Guo et al. investigated the SO_2_ tolerance of Fe_2_O_3_/SBA-15 catalysts with different pore sizes and found that the decomposition temperature of NH_4_HSO_4_ on the catalyst surface decreased by 40 °C when the pore size of the SBA-15 support increased from 4.8 to 11.8 nm, due to the fact that a larger pore size can produce a higher vapor pressure, thus facilitating the decomposition of NH_4_HSO_4_ [30]. Ma et al. also found that the hollow-structured CeO_2_–TiO_2_ catalyst exhibited a better SO_2_ resistance when it had a larger cavity [31].

At present, the MnO_x_–CeO_2_–TiO_2_ catalysts are prepared by several approaches, including the hydrothermal [26], sol–gel [24], wet impregnation [32] and co-precipitation methods [33], which show good low-temperature NH_3_–SCR performance. However, the catalytic activities in the presence of SO_2_ are still limited, probably due to their relatively small pore size. In this work, Mn–Ce–Ti–O composite aerogels with larger mesoporous pore sizes were prepared via a one-pot sol–gel method followed by calcination at different temperatures from 400 to 700 °C. The NH_3_–SCR catalytic activities with and without SO_2_ and H_2_O were investigated. The mechanisms were discussed based on the characterization results of X-ray diffraction (XRD), N_2_ adsorption, X-ray photoelectron spectroscopy (XPS), H_2_–temperature programmed reduction (H_2_–TPR), NH_3_–temperature programmed desorption (NH_3_–TPD), and in-situ diffuse reflectance infrared Fourier transform spectra (DRIFTS).

## 2. Materials and Methods

### 2.1. Catalyst Preparation

The Mn–Ce–Ti–O composite aerogel was prepared via a one-pot sol-gel method by using propylene oxide as a network gel inducer and ethyl acetoacetate as a complexing agent. The molar ratios of (Ti + Mn + Ce) to propylene oxide and ethyl acetoacetate were 1:5.5 and 1:0.3, respectively. Specific steps were as follows. Ethyl acetoacetate was added dropwise to 95 mL of anhydrous ethanol at a rate of 1 drop/s under magnetic stirring, and the mixed solution was put in an ice bath. Then, 0.1 mol of TiCl_4_ was added dropwise to the above solution by a syringe, and 0.04 mol of Mn(NO_3_)_2_ 4H_2_O and 0.007 mol of Ce(NO_3_)_3_·6H_2_O were added with consecutive stirring until they were completely dissolved. Finally, propylene oxide was added dropwise under continuous stirring for 30 min to obtain a yellow transparent sol. The sol was poured into an ampule (30 mL), sealed, aged in a 30 °C water bath for 2 days, and then aged in a 70 °C water bath for 5 days to obtain a wet gel. The wet gel was subjected to solvent displacement with n-hexane for 12 h, followed by supercritical drying in n-hexane at 240 °C and 6.0 MPa for 1 h with a heating rate of 1 °C min^−1^·to obtain the precursor of Mn–Ce–Ti–O composite aerogel. The precursor aerogel was calcined at 400, 500, 600, and 700 °C for 3 h with a heating rate of 5 °C min^−1^, respectively, to obtain the Mn–Ce–Ti–O composite aerogels, which were denoted as MCTO-x, where x represents the calcination temperatures.

### 2.2. Catalyst Characterization

The thermal stability of the MCTO-0 was tested by thermogravimetry-differential scanning calorimetry (TG–DSC) on a Netzsch STA 449 analyzer under air from room temperature to 800 °C with a heating rate of 10 °C min^−1^. The XRD patterns of samples were examined on a D/max 2200PC diffractometer using Cu Kα radiation as the source (λ = 0.15406 nm) with a 2θ range of 5–80° at a scan rate of 8°·min^−1^. The N_2_ adsorption-desorption curves were tested to analyze the specific surface area and pore texture of the samples at 77 K on a Micrometrics ASAP 2020 apparatus. Prior to each test, the samples were degassed at 200 °C for 12 h under vacuum. The specific surface area was calculated using the Brunauer–Emmett–Teller (BET) equation, and the pore size distribution and pore volume were calculated based on the density function theory (DFT) model. The morphology of samples was observed by scanning electron microscopy (SEM) with a FEI Quanta 200 FEG microscope. The XPS spectra of the samples were obtained on a Thermo Fisher Scientific ESCALAB 250Xi system using Al Kα as the radiation source at 300 W under ultrahigh vacuum (8 × 10^−8^ Pa). The C1s peak (284.8 eV) was used as a standard to calibrate the precise binding energy of Mn 2p, Ce 3d, Ti 2p, and O 1s. The H_2_–TPR and NH_3_–TPD experiments were performed on a Micrometrics AutoChem II 2920 instrument; 100 mg of samples were firstly pretreated at 200 °C for 1 h in an Ar atmosphere with a total flow rate of 30 mL·min^−1^, and then cooled to 50 °C. For H_2_–TPR, the pretreated catalysts were heated to 800 °C in a 10 vol. % H_2_/Ar mixture gas (30 mL·min^−1^) at a heating rate of 10 °C min^−1^. The H_2_ consumption of the reduction process was recorded by a thermal conductivity detector (TCD). For NH_3_–TPD, the pretreated samples were placed in a mixture gas of NH_3_/Ar (10 vol. % of NH_3_) with a total flow rate of 50 mL·min^−1^ for 1 h until saturation to ensure a sufficient adsorption of NH_3_ on the catalyst surfaces. Then pure N_2_ was blown in for 30 min to remove the non-adsorbed NH_3_. Finally, the samples were placed in an He atmosphere with a flow rate of 50 mL·min^−1^ to carry out NH_3_ desorption at a heating rate of 10 °C min^−1^ from 50 °C to 800 °C to obtain the NH_3_–TPD curves. The in-situ DRIFTS were collected on an FTIR spectrometer (Thermo Fisher Nicolet iZ10) equipped with a smart collector and an MCT/A detector that was cooled by liquid nitrogen. Before each test, the catalysts were pretreated at 350 °C for 2 h under N_2_ with a flow rate of 50 mL·min^−1^ and cooled down to 200 °C. The background spectrum was collected in N_2_ atmosphere and automatically deducted from the sample spectrum. In the transient NH_3_–SCR performance experiment, the MCTO-600 catalyst was firstly exposed to 500 ppm of NH_3_ (or 500 ppm of NO + 5 vol. % O_2_) stream for 30 min at 200 °C, then purged with He for 30 min, and finally subjected to 500 ppm of NO + 5 vol. % O_2_ (or 500 ppm of NH_3_) to obtain the time-varying DRIFTS. All spectra were collected by accumulating 32 scans at a resolution of 4 cm^−1^.

### 2.3. Catalytic Activity Measurement

The NH_3_–SCR activity of catalysts was evaluated by a fixed-bed reactor (id: 7.5 mm), in which 0.5 mL of catalysts with 40–80 mesh was fixed by quartz sand (40–60 mesh) and glass wool. The feed-gas consisted of 500 ppm NO, 500 ppm NH_3_, 5 vol. % O_2_, 5 vol. % H_2_O (when used), 100 ppm SO_2_ (when used), and N_2_ balance, with a total flow rate of 120 mL·min^−1^, corresponding to the gas hourly space velocity (GHSV) of 14,400 h^−1^. The catalyst reaction temperature was controlled from 120 to 320 °C with a heating rate of 5 °C min^−1^ from 20 °C to the reaction temperatures and held for 30 min to reach a steady state for data collection. The concentrations of NO and NO_2_ were measured by the chemiluminescence method with an Eco Physics CLD 62s NO/NO_x_ analyzer, and the concentration of N_2_O was detected by gas chromatography using a Techcomp GC 7900 apparatus.

The NO conversion (x) and the N_2_ selectivity (S) were calculated using the Equations (1) and (2), respectively [6,9].

(1) $$x=\frac{{\left[NO\right]}_{in}-{\left[NO\right]}_{out}}{{\left[NO\right]}_{out}}\times 100\%$$

(2) $$S=\left(1-\frac{{\left[N_{2}O\right]}_{out}}{{\left[NO_{x}\right]}_{in}-{\left[NO_{x}\right]}_{out}}\right)\times 100\%$$

where [*NO*]*_in_*signifies the inlet concentration of NO, [*NO*]*_out_* stands for the outlet concentration of NO, and [*N*_2_*O*]*_out_* is the outlet concentration of N_2_O.

In addition, in order to better evaluate the catalytic activity of Mn–Ce–Ti–O aerogels, a steady state kinetics investigation on these catalysts was conducted. The calculation of the reaction rate constant *K* (cm^3^·g^−1^·s^−1^) is based on the assumption that *[NH_3_]* is a zero-order reaction and *[NO]* is a first-order reaction [2,34]. The *K* is calculated by the Equation (3):

(3) $$K=\frac{-V\ln\left(1-x\right)}{w}$$

where *K* is the reaction rate constant (cm^3^·g^−1^·s^−1^), *V* means the total flow rate (mL·min^−1^) of the simulated flue gas, *w* represents the mass (g) of the catalyst, and *x* is the NO conversion. The apparent activation energy could be obtained by using the Arrhenius equation, as given by Equation (4) as follows:

(4) $$K\;=\;K_{0}e^{\frac{-E_{a}}{RT}}$$

where *E_a_* represents the apparent activation energy of catalysts, which could be calculated from the slope of the *ln(K)* versus 1000/T curves.

To compare the intrinsic activity of the catalysts at different calcination temperatures, turnover frequency (TOF) was calculated using Equation (5) [14,35]. TOF represents the number of NO conversion at a single active site (Mn atom) per unit time; its value is a measure of the catalytic reaction rate on a catalyst. In general, the calculation of TOF requires the conversion of a reactant to be at a relatively low level (<15%) [36,37]. Reaction conditions were as follows: T = 160, 180, 200, or 220 °C, 500 ppm of NO, 500 ppm of NH_3_, 5 vol. % of O_2_, balance N_2,_ total flow rate = 240 mL·min^−1^, GHSV ≈ 200,000 h^−1^.

(5) TOF=(PVRT)xw×βMnMMn

where *P*, *V*, *R*, *T*, *x*, *w*, *β_Mn_*, and *M_Mn_* represents the standard atmospheric pressure (101 KPa), the NO flow rate (0.12 mL·min^−1^), the gas constant (8.314 J·mol^−1^·K^−1^), the reaction temperature (K), the NO conversion at each temperature, the mass of the catalyst (0.02 g), the atomic percentage of Mn on the surface of the catalyst by XPS, and the relative atomic mass of Mn (54.94 g·mol^−1^), respectively.

## 3. Results and Discussion

### 3.1. Catalyst Characterization

**Figure 1:**
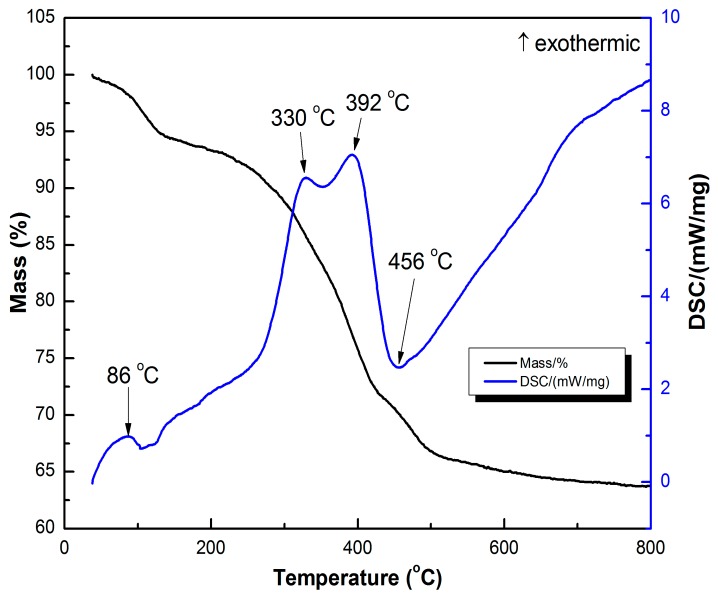
Thermogravimetry–differential scanning calorimetry (TG–DSC) curve of sample MCTO-0.

The TG–DSC curve of the MCTO-0 aerogel under air was tested to study its thermal decomposition behavior, which contained three mass loss stages, as shown in Figure 1. The first mass loss stage below 120 °C with an exothermic peak at 86 °C was due to the desorption of adsorbed water. The second mass loss stage in the range of 120–400 °C with two exothermic peaks at 330 °C and 392 °C was detected, which could be attributed to the removal of organic matter and the decomposition of metal oxide precursor. In addition, an endothermic peak at 456 °C was observed for the third mass loss step from 400 to 600 °C. There were mass loss and mass gain involved at this step. The former was caused by carbon burn-off in the temperature range of 400–600 °C, which was 8.2 wt % (from XPS results), and the latter was from the oxidation of Mn_3_O_4_ to Mn_2_O_3_ (2Mn_3_O_4_ + 1/2O_2_ = 3Mn_2_O_3_), which was 3.5 wt %. Therefore, a mass loss was observed at this step.

**Figure 2:**
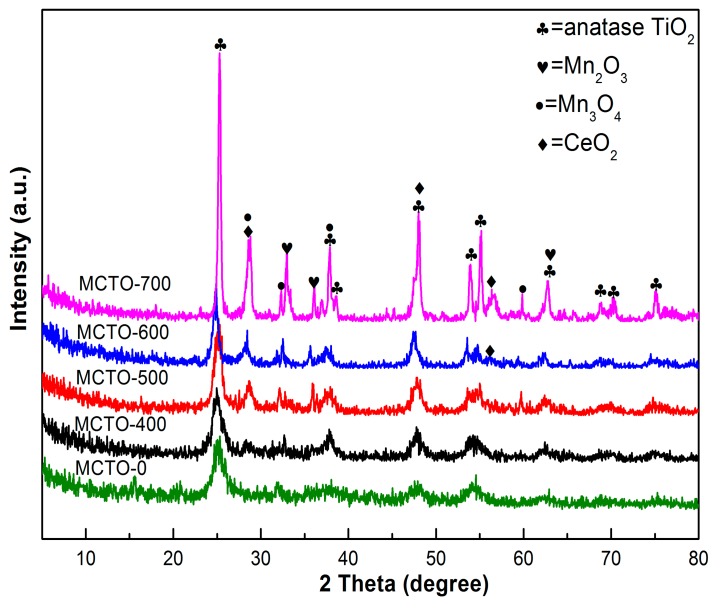
XRD patterns of MCTO-0, MCTO-400, MCTO-500, MCTO-600, and MCTO-700 catalysts.

The XRD patterns of the MCTO-0, MCTO-400, MCTO-500, MCTO-600, and MCTO-700 are shown in Figure 2. The diffraction peaks at 25.3°, 37.8°, 38.6°, 48.1°, 51.0°, 55.1°, 62.7°, 68.9°, 70.3°, and 75.3° were observed for MCTO-0 sample, which were attributed to anatase TiO_2_ (JCPDS 21-1272) [11]. The characteristic peaks of the Mn_3_O_4_ phase (JCPDS 24-0734) at 28.9°, 32.3°, 38.0°, and 59.8° and the Mn_2_O_3_ phase (JCPDS 33-0900) at 32.3°, 35.6°, and 62.3° were detected for the calcined products [38]. In addition, the diffraction peaks for the CeO_2_ phase with a cubic fluorite structure (JCPDS 34-0394) at 28.8°, 48.2°, and 56.7° appeared for samples MCTO-600 and MCTO-700 [4]. The mean crystallite sizes of the above different phases were calculated using the Scherrer formula, as shown in Table 1; it could be seen that the increase of calcination temperature resulted in the gradual increase in crystallite sizes for the above metal oxides. Significantly, according to the results of below XPS and H_2_–TPR, MnO_2_ species were found on the surface of all samples. However, one could not see any standard peaks ascribed to MnO_2_ in XRD patterns, suggesting that they were highly dispersed on the catalyst surface, which could be beneficial to the NH_3_–SCR activity.

**Figure 3:**
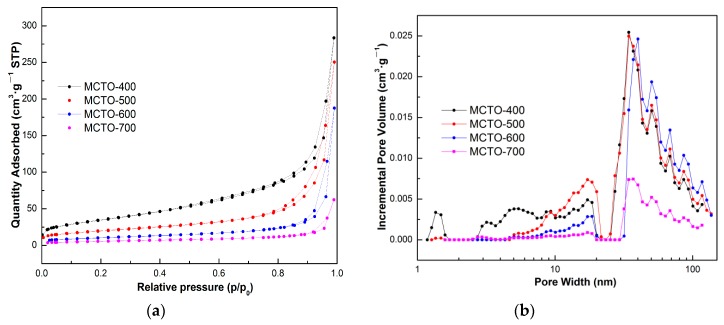
N_2_ adsorption/desorption isotherms (**a**) and the corresponding pore size distributions (**b**) of MCTO-400, MCTO-500, MCTO-600, and MCTO-700.

To gain insight into the pore structure of the obtained catalysts, the N_2_ adsorption-desorption isotherms of the catalysts were examined, as shown in Figure 3a. All the isotherms exhibited a typical IV-type curve with an H3 hysteresis loop, suggesting the existence of mesoporous pores [39]. In addition, a high nitrogen uptake at high relative pressure over 0.95 was observed for all the samples, indicating the existence of macropores [40], in accordance with the bimodal pore size distributions displayed in Figure 3b. The smaller pores may have been formed by the primary nanoparticles and the larger pores by the secondary particles as a result of aggregation of the primary nanoparticles. With increases in the calcination temperature, the sintering of primary nanoparticles occurred, leading to their gradual disappearance. The secondary particles were relatively stable with increasing calcination temperature, but an apparent decrease of larger pores formed by secondary particles was found at 700 °C, indicating that sintering of the secondary particles occurred. This was why the NH_3_–SCR activity of the MCTO-700 was lower than that of the MCTO-600. 

It can be seen from the Table 2, the BET and pore volume of the catalysts decreased gradually with increasing the calcination temperature. And it is noted that the BET surface area of the MCTO-600 was not the highest among the catalysts, indicating that the specific surface area may not play an important role on the NH_3_–SCR activity of MCTO-x catalysts [12]. Furthermore, the mean pore size of the MCTO-600 was largest among the catalysts due to the reduction of smaller pores below 20 nm while retaining larger pores above 20 nm.

**Figure 4:**
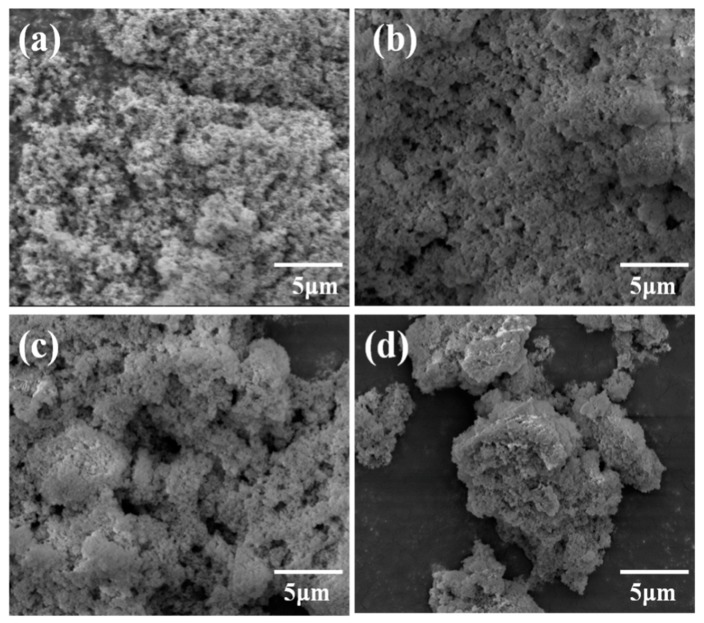
SEM images of MCTO-400 (**a**), MCTO-500 (**b**), MCTO-600 (**c**), and MCTO-700 (**d**).

Figure 4 shows the SEM images of Mn–Ce–Ti–O composite aerogels obtained at different calcination temperatures. It can be seen that the samples were composed by interconnected particles with a porous structure, and the particle size grew gradually with the increase of calcination temperature, showing an obvious particle agglomeration for the sample MCTO-700, in accordance with the above XRD and N_2_ adsorption–desorption results.

**Figure 5:**
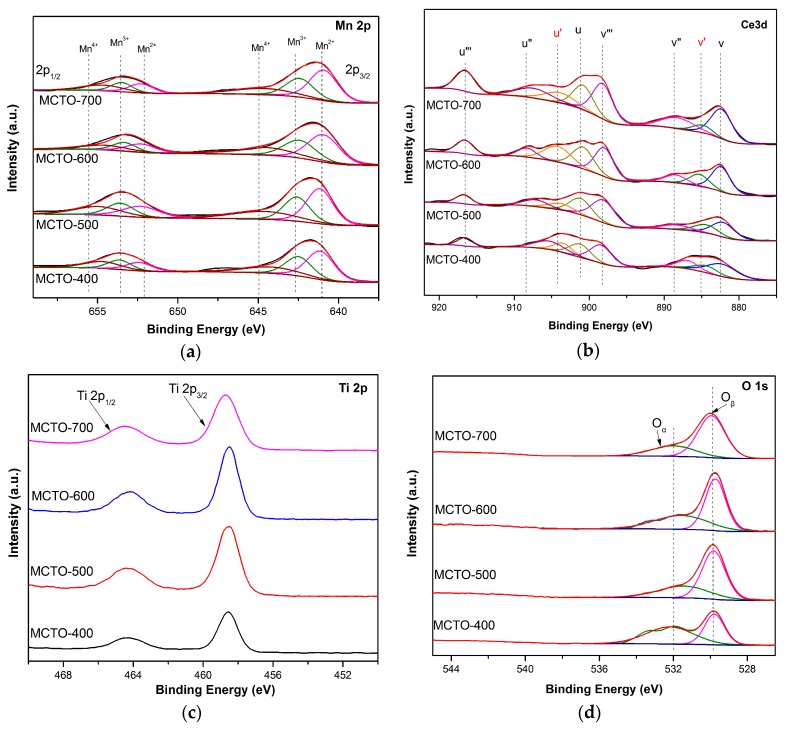
XPS spectra of the MCTO-400, MCTO-500, MCTO-600, and MCTO-700 catalysts: (**a**) Mn 2p, (**b**) Ce 3d, (**c**) Ti 2p, and (**d**) O 1s.

XPS technology was used to study the surface atomic content and chemical states of Mn, Ce, Ti, and O. As shown in Figure 5a, the Mn 2p spectra contained two main peaks at 653.5–653.8 eV and 641.5–641.7 eV, which could be attributed to Mn 2p1/2 and Mn 2p3/2, respectively. The deconvolution of Mn 2p1/2 peak could obtain three peaks assigned to Mn^2+^ (652.4 ± 0.2 eV), Mn^3+^ (653.8 ± 0.3 eV), and Mn^4+^ (654.9 eV ± 0.4), The Mn 2p3/2 could also be divided into three peaks ascribed to Mn^2+^ (641.1 ± 0.2 eV), Mn^3+^ (642.6 ± 0.3 eV), and Mn^4+^ (644.1 ± 0.4 eV) [20,21]. The XPS spectra of Ce 3d in Figure 5b could be divided into eight characteristic peaks, which were labeled as u (900.8–902.0 eV), u′ (903.3–904.0 eV), u″ (906.8–908.0 eV), u‴ (916.0–917.0 eV), v (882.0–882.4 eV), v′ (884.3–885.0 eV), v″ (887.9–888.4 eV), and v‴ (898.0–898.4 eV), The two peaks, u′ and v′, were attributed to Ce^3+^ species, whereas another six peaks belonged to Ce^4+^ species [14,38]. Two peaks, at ~464.5 eV and ~458.7 eV, were observed for the XPS spectra of Ti 2p, as shown in Figure 5c, which were attributed to Ti 2p3/2 and Ti 2p1/2, respectively, representing the characteristic Ti^4+^ species [11,14]. The XPS spectra of O1s in Figure 5d exhibited two doublet peaks at 531.1–531.8 eV and 529.3–529.8 eV, which were assigned to the surface chemisorbed oxygen (denoted as O_α_) such as defect-oxide (O_2_^2−^ or O^−^) and hydroxyl groups, and the lattice oxygen O^2−^ (denoted as O_β_), respectively [11,41]. The relative contents of different valence states could be calculated from the ratios of peak areas of XPS spectra, as listed in Table 3.

As can be seen from Table 3, with the increase of the calcination temperature, the relative atomic ratios of Mn^4+^/Mn on the surface of MCTO-x catalyst increased firstly and then decreased, and the highest ratio of Mn^4+^/Mn was present on the surface of the MCTO-600, which showed that an appropriate calcination temperature was favorable for the formation of Mn^4+^. As the contents of the surface Mn element for the four samples were different, the absolute surface Mn^4+^ contents for the MCTO-400, MCTO-500, MCTO-600, and MCTO-700 were 1.40%, 1.53%, 1.35%, and 1.19%, respectively. This difference could be caused by sintering and motion of primary nanoparticles in calcination, which caused different accessibilities for X-rays for different samples. It has been proved that Mn^4+^ species and their redox cycle can accelerate the conversion of NO to NO_2_, thereby enhancing the low-temperature NH_3_–SCR performance via the “fast-SCR” path (2NH_3_ + NO + NO_2_ = 2N_2_ + 3H_2_O) [18], which displays a reaction rate 10 times higher than that of the standard SCR process [42]. The proportion of Ce^3+^/Ce and absolute Ce^3+^ content on the surface of the MCTO-600 reached 24.0% and 0.41%, respectively, which were the highest among the four catalysts investigated. Ce^3+^ could generate a charge imbalance to form oxygen vacancies and unsaturated chemical bonds on the surface of the catalysts and promote the migration of oxygen from the bulk to the surface, thus accelerating the oxidation of NO to NO_2_ to facilitate the SCR reaction [22,23]. It has been widely reported that O_α_ species are more active than O_β_ species due to their higher mobility, and a high O_α_/(O_α_ + O_β_) ratio favors the oxidation of NO to NO_2_ in NH_3_–SCR to enhance the low-temperature activity of the catalyst [18,43]. It is noted that the percentage of O_α_ species was the highest for the MCTO-400 among the catalysts, which may be due to the smallest crystallite sizes at the lowest calcination temperature. The highest ratio of O_α_/(O_α_ + O_β_) on the surface of the MCTO-400 catalyst corresponded to the most abundant Brønsted acid sites from the NH_3_–TPD results. However, the MCTO-400 had the lowest NH_3_–SCR performance among the four catalysts investigated. In addition, when the calcination temperature reached 700 °C, O_α_ decreased sharply, which may be due to the growth of crystallite size. These suggest that the number of surface chemisorbed oxygen might not the determining fact for the NH_3_–SCR activity in this work.

**Figure 6:**
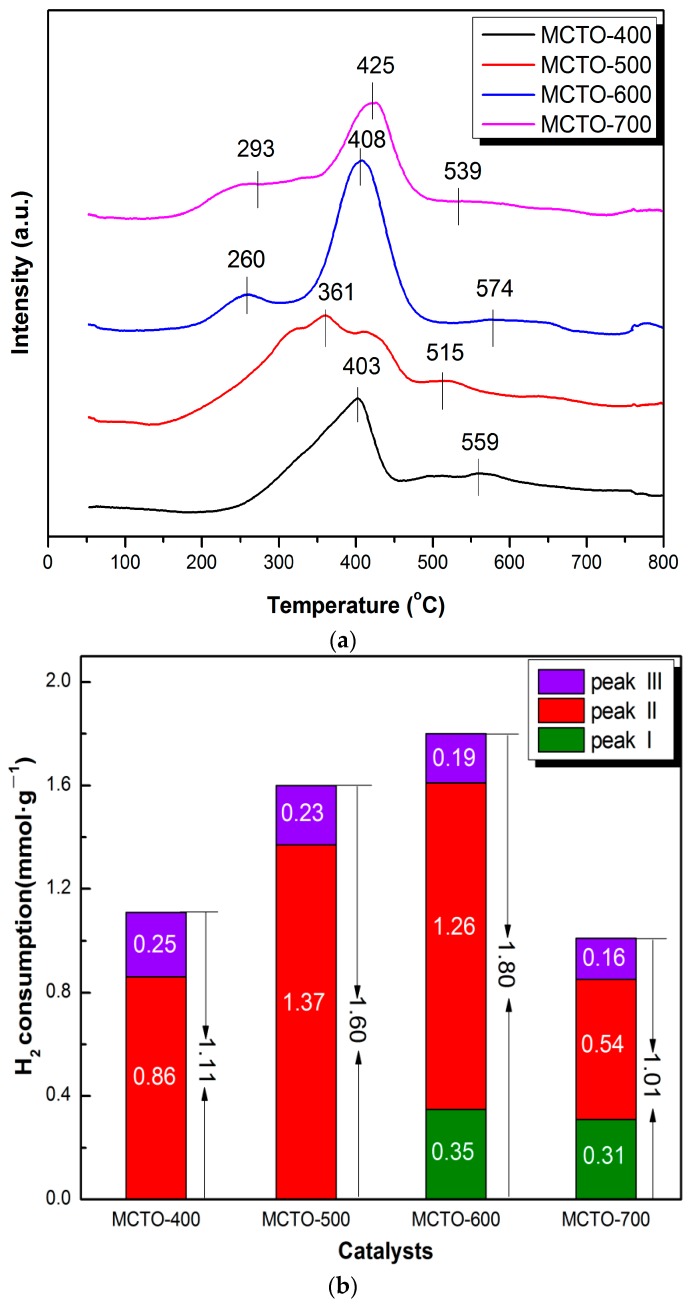
H_2_–temperature programmed reduction (TPR) profiles (**a**) and the corresponding H_2_ consumption (**b**) of the MCTO-400, MCTO-500, MCTO-600, and MCTO-700 catalysts.

The H_2_–TPR technique was used to explore the reduction performance of the MCTO-x catalyst, and the obtained H_2_–TPR profiles and corresponding H_2_ consumption are shown in Figure 6a,b. The peak at 200–300 °C (denoted as peak “I”) was assigned to MnO_2_→Mn_2_O_3_ for the MCTO-600 and MCTO-700. The peak at 300–500 °C (denoted as peak “II”) corresponded to Mn_2_O_3_→Mn_3_O_4_ for all samples. For the MCTO-400 and MCTO-500, the reduction at 200–300 °C corresponding to MnO_2_→Mn_2_O_3_ was not well resolved with no apparent peak found. This could be caused by a smaller number of the high valence Mn^4+^ at a lower calcination temperature. The peak at 500–600 °C (denoted as peak “III”) was attributed to the reduction of CeO_2_ to Ce_2_O_3_ or surface Mn–O–Ce species [22]. As shown in Figure 6b, as the calcination temperature increased, the total H_2_ consumption of the catalysts increased firstly and then decreased. The MCTO-600 had the best reducibility, but its absolute surface Mn^4+^ content was not the highest, which might be due to the fact that the accessibility of the X-ray for the surface of samples by XPS was different from that of the reactants, and that the former had a higher accessibility than the latter. With increases in the calcination temperature, the number of the high valence ions can increased, and therefore the total H_2_ consumption can increase. The decrease of the total H_2_ consumption beyond 600 °C can be caused by the growth of crystallite size due to sintering and the decrease of the number of available reducible species. As the total H_2_ consumption of the MCTO-600 exhibited the largest of 1.80 mmol g^−1^, therefore, its low-temperature NH_3_–SCR activity was the highest among the four samples, indicating that the reducibility of active species favored the improvement of the NH_3_–SCR activity [14,18,38].

**Figure 7:**
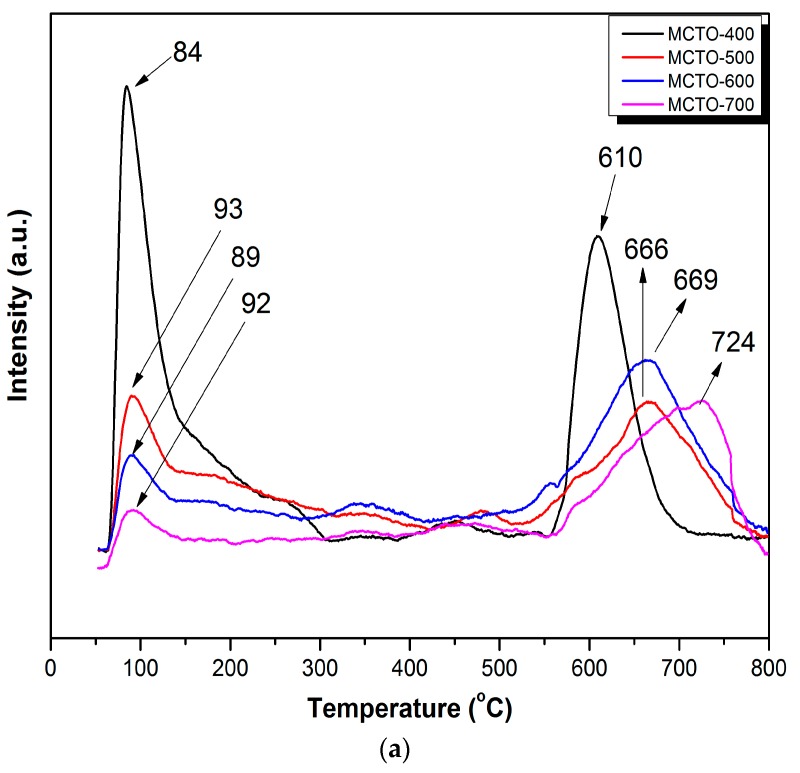

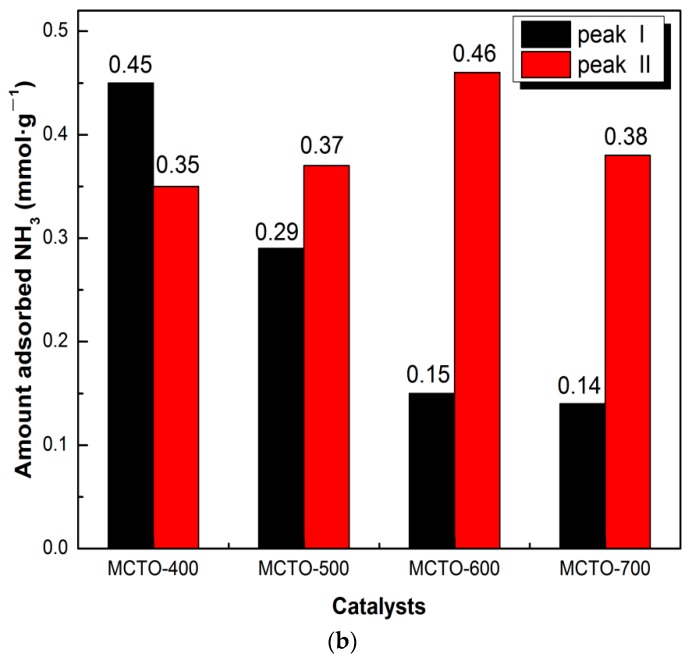
NH_3_–TPD profiles (**a**) and the corresponding amount of adsorbed NH_3_ (**b**) of the MCTO-400, MCTO-500, MCTO-600, and MCTO-700 catalysts.

It has been generally confirmed that the adsorption and activation of NH_3_ on the acid sites of the catalyst surface is a key step in the NH_3_–SCR, so the NH_3_–TPD test was used to study the number and the strength distribution of acid sites over the catalysts. As shown in Figure 7a, two evident peaks could be observed from the TPD profiles, namely peak “I” at 70–450 °C, ascribed to the NH_3_ desorption by weak and medium acid sites, and peak “II” at 450–800 °C, due to the desorption of NH_3_ at strong acidic sites. It was reported that the NH_3_ molecules coordinated on the Lewis acid sites show a stronger thermal stability than the NH_4_^+^ ions formed on the Brønsted acid sites [44]. Thus, it can be inferred that the peak “I” belonged to the desorption of NH_3_ on the Brønsted acid sites, and the peak “II” was attributed to the desorption of NH_3_ from the Lewis acid sites. It can be seen from Figure 7b that the amount of the surface Brønsted acid sites of MCTO-x gradually decreased with the increase of calcination temperature, and the order was as follows: MCTO-400 (0.45 mmol·g^−1^) > MCTO-500 (0.29 mmol·g^−1^) > MCTO-600 (0.15 mmol·g^−1^) > MCTO-700 (0.14 mmol·g^−1^). With increases in the calcination temperature, the MCTO-600 exhibited the highest amount of adsorbed NH_3_ on Lewis acid sites (0.46 mmol·g^−1^), which had a stronger ability to adsorb and activate NH_3_ than Brønsted acid sites to facilitate the NH_3_–SCR process [2,45]. However, when the calcination temperature reached 700 °C, the amount of Lewis acid sites on the catalyst surface also began to decrease, which indicated that the higher calcination temperature led to the reduction of both the Brønsted and Lewis acid sites, causing weak NH_3_ adsorption and activation. This might have been caused by the growth of crystallite size due to sintering and the decrease of the number of both the Brönsted and Lewis acid sites. The Brønsted acid sites were contributed to by –OH groups linked to Mn and Ce ions with different valences [46], which was why the amount of the surface Brønsted acid sites decreased with increases in the calcination temperature due to enhanced dehydration at high temperature. As MnO_2_ was in an amorphous state as indicated by XRD, the Brønsted acid sites were contributed to by Mn^4+^. The Lewis acid sites were mainly contributed to by the oxides of Mn^3+^, Mn^2+^, and Ce^3+^ in a dehydrated state [47]. As Mn_2_O_3_ and Mn_3_O_4_ were in crystalline states while Ce_2_O_3_ was in an amorphous state as indicated in XRD, the Lewis acid sites were mainly contributed to by Ce^3+^. The MCTO-600 had the highest amount of Ce^3+^, so it had the most abundant Lewis acid sites.

**Figure 8:**
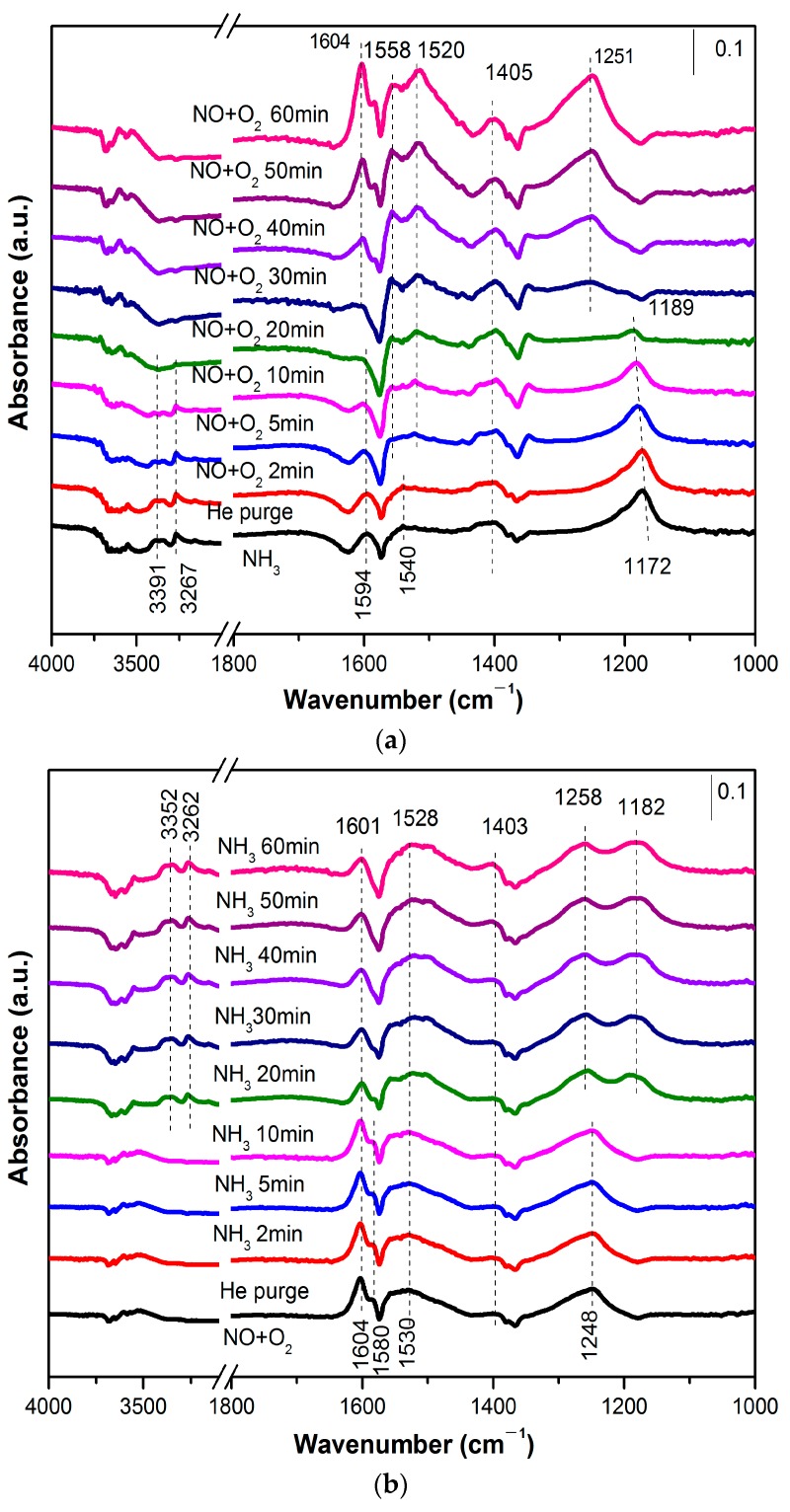
In-situ diffuse reflectance infrared Fourier transform spectra (DRIFTS) of NO + O_2_ reacted with pre-adsorbed NH_3_ species over MCTO-600 (**a**). In-situ DRIFTS of NH_3_ reacted with pre-adsorbed NO + O_2_ over MCTO-600 (**b**). Reaction conditions: T = 200 °C, 500 ppm of NO (when used), 500 ppm of NH_3_ (when used), 5 vol. % of O_2_ (when used), balance N_2_.

The in-situ DRIFTS was examined for the sample MCTO-600 in order to study the catalytic reaction mechanism of the Mn–Ce–Ti–O composite aerogel. During the experiment, NH_3_ was firstly pre-adsorbed at 200 °C for 30 min, then purged with He for 30 min, and finally NO + O_2_ was passed in for different times to obtain the results, as shown in Figure 8a. After the introduction of NH_3_, some NH_3_ species appeared on the surface of the MCTO-600 catalyst, including the coordinated NH_3_ on the Lewis acid sites (1172, 1594, 3267, 3391 cm^−1^) [48,49], the NH_4_^+^ adsorbed on Brønsted acid sites (1405 cm^−1^), and –NH_2_ (1540 cm^−1^) [48,50]. From the different intensities of the Lewis and Brønsted acid species, it was found that Lewis acid sites were dominant in the MCTO-600, in accordance with the NH_3_–TPD results. When NO + O_2_ was introduced, the peak intensity of NH_3_ (L) species on the catalyst surface gradually decreased and disappeared after 20 min. The bands of NH_3_ (B) species showed no significant change in strength after 60 min of NO + O_2_ introduction, indicating that it was Lewis, not Brønsted, acidity that contributed mainly to the NH_3_–SCR reaction under this condition; so the largest amount of Lewis acid sites on the surface of the MCTO-600 catalyst played an important role for its highest NH_3_–SCR activity. The peaks ascribed to the adsorbed NO appeared after 20 min, while the peaks attributed to NH_3_ (L) species disappeared. The –NH_2_ species, which may be the amides produced by the dehydrogenation of adsorbed NH_3_, completely disappeared within 2 min, because they could react directly with gaseous NO to form the intermediate state NH_2_NO, and finally rapidly decomposed into N_2_ and H_2_O [2,19]. This suggests that adsorbed NH_3_ (L) species reacted with gaseous NO and O_2_, indicating that the Eley–Rideal (E–R) mechanism [2] was involved in the NH_3_–SCR process. After introduction of NO + O_2_ for 30 min, the surface of the catalyst was covered by adsorbed NO_x_ species, including bridging nitrate species (1251 cm^−1^) [11], monodentate nitrate species (1520 cm^−1^) [51], bidentate nitrate species (1558 cm^−1^) [15], and gaseous NO_2_ species (1604 cm^−1^) [48]. This suggests that the Langmuir–Hinshelwood (L–H) mechanism was also possible. 

Similarly, the transient SCR reaction of NH_3_ with pre-adsorbed NO + O_2_ on the MCTO-600 catalyst was recorded by in-situ DRIFTS, and the results of the reaction at different times are shown in Figure 8b. After the MCTO-600 catalyst was pretreated by NO + O_2_, bridged nitrate species (1248 cm^−1^), monodentate nitrate species (1403 and 1530 cm^−1^), bidentate nitrate species (1580 cm^−1^), and gaseous NO_2_ (1604 cm^−1^) species appeared on the catalyst surface. The co-existence of NH_3_-adsorbed peaks (coordinated NH_3_–L species at 1182, 3262, and 3352 cm^−1^) and NO-adsorbed peaks (1403, 1528, and 1601 cm^−1^) was found after NH_3_ was introduced for 10 min, indicating that the Langmuir–Hinshelwood (L–H) mechanism was also involved in the NH_3_–SCR process [2]. When NH_3_ was introduced for less than 10 min, no peaks ascribed to adsorbed NH_3_ were found. This could be accounted for by the fact that the Lewis acid sites were abundant, which diluted the NH_3_–adsorbed species, and that fast reaction between the adsorbed NH_3_ and adsorbed NO occurred, leading to a low concentration of NH_3_–adsorbed species that were out of the limits of DRIFTS. This implies that the Langmuir–Hinshelwood (L–H) mechanism was involved in the NH_3_–SCR process as the activation energy for the Langmuir–Hinshelwood (L–H) mechanism was lower than that of the Eley–Rideal (E–R) mechanism [52].

In addition, a new band at 1258 cm^−1^ may belong to the surface ammonium nitrate, which originated from the dimerization of NO_2_ and the continuous reaction with NH_3_ and H_2_O [19,53]. It has been confirmed that NH_4_NO_3_ is also an important intermediate component in the NH_3_-SCR process, along the following reaction route: NH_4_NO_3_ + NO→NH_4_NO_2_ + NO_2_, NH_4_NO_2_→N_2_ + 2H_2_O [54]. The presence of NO_2_ species demonstrated that a high ratio of Mn^4+^/Mn facilitated the oxidation of NO to NO_2_. 

### 3.2. Catalytic Performance 

**Figure 9:**
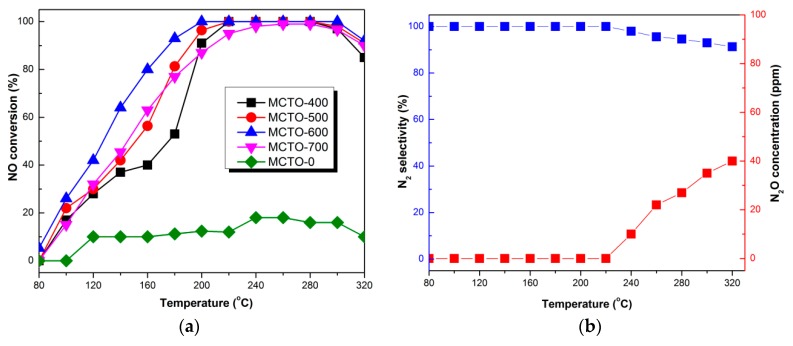
NH_3_–selective catalytic reduction (SCR) activity of MCTO-0, MCTO-400, MCTO-500, MCTO-600, and MCTO-700 catalysts (**a**). N_2_ selectivity of MCTO-600 (**b**). Reaction conditions: 500 ppm of NO, 500 ppm of NH_3_, 5 vol. % of O_2_, balance N_2_, gas hourly space velocity (GHSV) = 14,400 h^−1^.

The NH_3_–SCR performance of Mn–Ce–Ti–O composite aerogels with different calcination temperatures is shown in Figure 9a. Almost no catalytic activity was observed for the uncalcined composite aerogel in the temperature range of 80–320 °C, showing only about 10% of NO conversion. However, the calcined products exhibited excellent low-temperature catalytic activity, and it was shown that the NO conversion increased with increases in the calcination temperature up to 600 °C and then decreases with further increases in the calcination temperature. The MCTO-600 exhibited the most excellent catalytic activity, achieving more than 80% of NO conversion at 160–320 °C and nearly 100% from 200–300 °C. Figure 9b shows the outlet N_2_O concentration and N_2_ selectivity on the MCTO-600 catalyst. One hundred percent of N_2_ selectivity was observed in the temperature range of 120–220 °C, whereas N_2_O was detected when the reaction temperature was above 220 °C, which may be because a part of NH_3_ was oxidized at high temperatures [26]; nevertheless, more than 90% of N_2_ selectivity was obtained at 240–320 °C.

### 3.3. Kinetics Parameters

**Figure 10:**
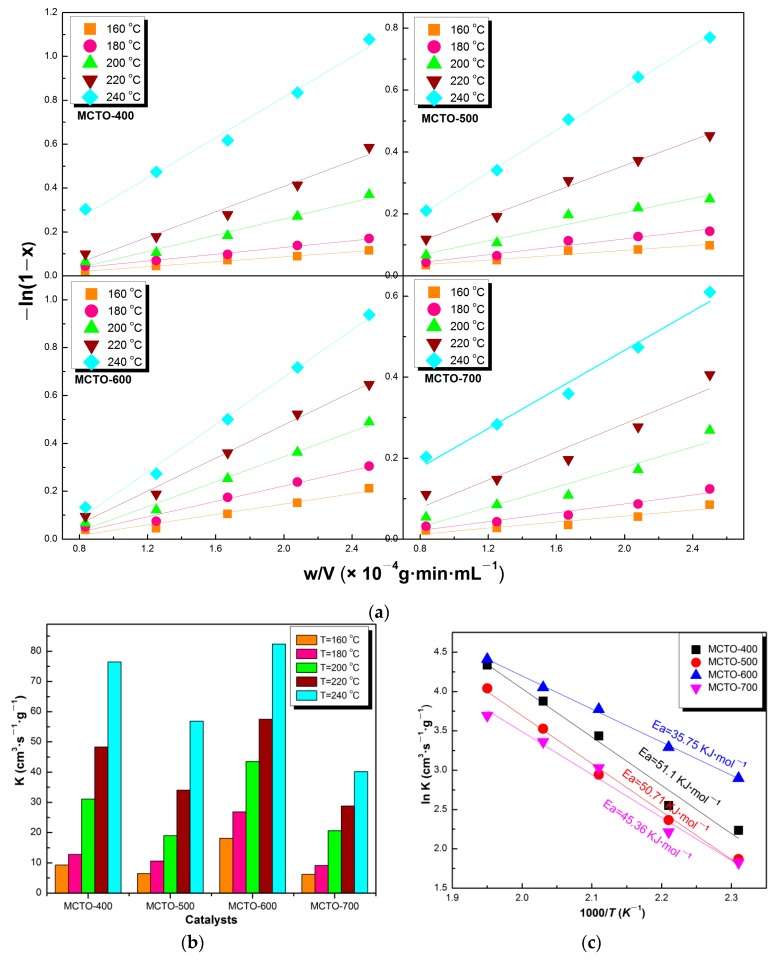
−ln(1 − x) versus w/V plots (**a**), reaction rate constant (**b**), and Arrhenius plots (**c**) of MCTO-400, MCTO-500, MCTO-600, and MCTO-700.

As shown in Figure 10a–c, the calcination temperature had a certain influence on the reaction rate constant and *E_a_* of the obtained catalysts. With increases in temperature, the reaction rate constant increased gradually (Figure 10b), reaching the highest for the MCTO-600 at each temperature (18.16 cm^3^·s^−1^·g^−1^ at 160 °C, 26.90 cm^3^·s^−1^·g^−1^ at 180 °C, 43.55 cm^3^·s^−1^·g^−1^ at 200 °C, 57.54 cm^3^·s^−1^·g^−1^ at 220 °C, and 82.34 cm^3^·s^−1^·g^−1^ at 240 °C). Furthermore, as the calcination temperature increased, the *E_a_* of the catalyst decreased firstly and then increased as follows: MCTO-600 (35.75 kJ·mol^−1^) < MCTO-700 (45.36 kJ·mol^−1^) < MCTO-500 (50.71 kJ·mol^−1^) < MCTO-400 (51.41 kJ·mol^−1^) (Figure 10c), indicating the energy barrier of the NH_3_–SCR reactions on the MCTO-600 was the lowest, which was related to its best NH_3_–SCR performance. In addition, it should be mentioned that the *E_a_* of Mn–Ce–Ti–O composite catalysts prepared by this work were relatively lower compared with the other reported catalysts, such as the commercial V-W/Ti catalyst (73.9 kJ·mol^−1^) [10], Fe–ZSM-5 (54 kJ·mol^−1^) [5], and WO_3_/Fe_2_O_3_ (62–66 kJ·mol^−1^) [37].

**Figure 11:**
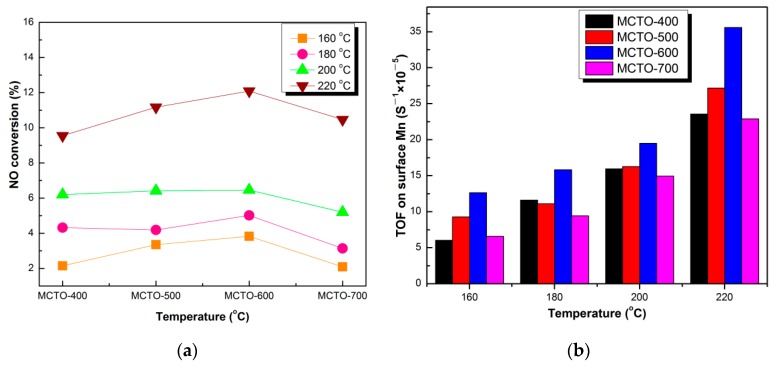
NH_3_–SCR performance (**a**) and the corresponding TOF (**b**) of MCTO-400, MCTO-500, MCTO-600, and MCTO-700 at different temperatures. Reaction conditions: 500 ppm of NO, 500 ppm of NH_3_, 5 vol. % O_2_, balance N_2,_ catalyst mass = 20 mg, flow rate = 240 mL·min^−1^, GHSV ≈ 200,000 h^−1^.

TOF was used to further compare the intrinsic activity of the catalysts at 160, 180, 200, and 220 °C, respectively, which was calculated based on the atomic percentage of Mn (the main active site) on the surface. As shown in Figure 11a,b, it can be seen that MCTO-600 presented the highest TOF value compared with that of MCTO-400, MCTO-500, and MCTO-700, which was 12.64 × 10^−5^·s^−1^ at 160 °C, 15.83 × 10^−5^·s^−1^ at 180 °C, 19.51 × 10^−5^·s^−1^ at 200 °C, and 35.57 × 10^−5^·s^−1^ at 220 °C, further demonstrating that NO conversion on the MCTO-600 was highest, in accordance with the results from the activation energy.

### 3.4. Influence of SO_2_ and H_2_O and Stability Test

**Figure 12:**
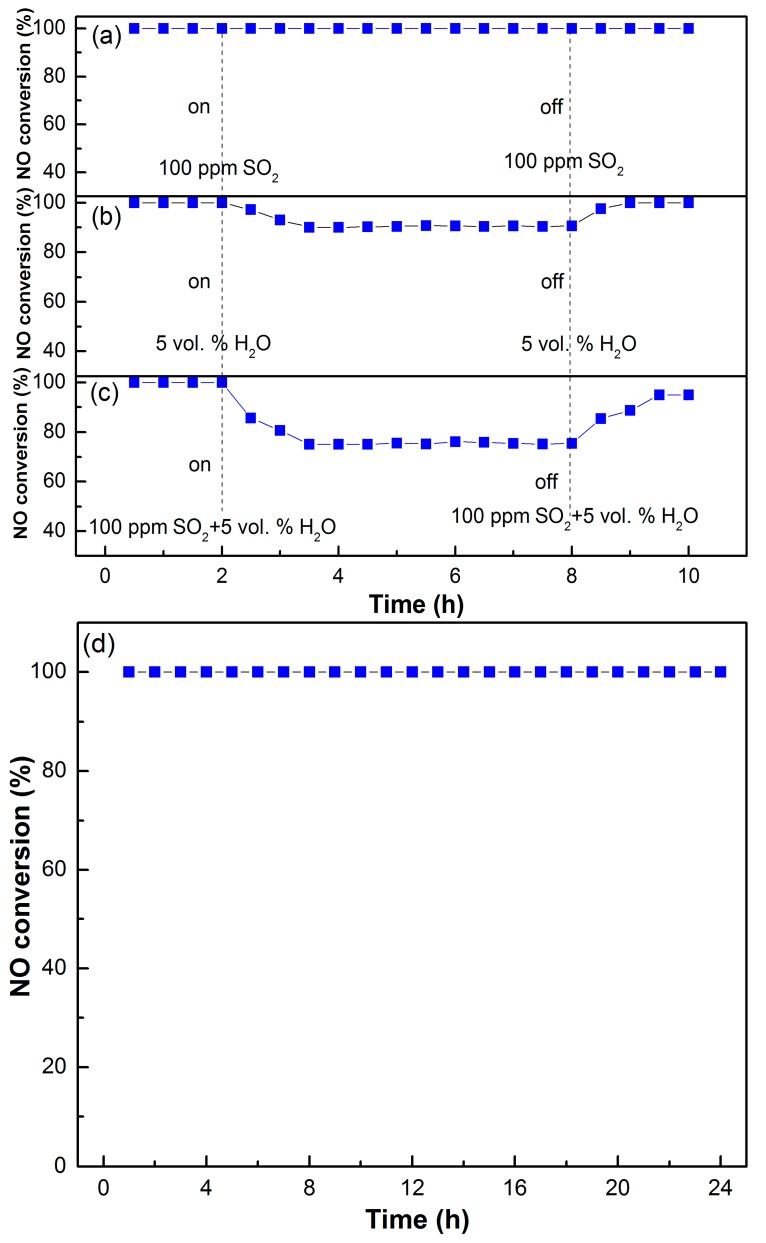
The influence of SO_2_ (**a**), H_2_O (**b**), and SO_2_ + H_2_O (**c**) on NO conversion and long-time stability test (**d**) of the MCTO-600 catalyst. Reaction conditions: T = 200 °C, 500 ppm of NO, 500 ppm of NH_3_, 5 vol. % of O_2_, 5 vol. % of H_2_O (when used), 100 ppm of SO_2_ (when used), and balance N_2_; GHSV = 14,400 h^−1^.

In actual industrial processes, some residual SO_2_ is present in the desulfurized flue gas, which may poison and deactivate the denitration catalysts at low temperature. Therefore, the SO_2_ tolerance performance of the MCTO-600 catalyst was further studied, as shown in Figure 12a. The NO removal efficiency was stabilized at 100% for approximately 2 h at 200 °C prior to the injection of SO_2_. When 100 ppm of SO_2_ was introduced into the simulated flue gas, the NO conversion of the catalyst remained nearly 100% within 6 h, which was unchanged for the next 2 h after the injection of SO_2_ was cut off, indicating the excellent SO_2_ resistance for the MCTO-600. It has been acknowledged that the TiO_2_ and CeO_2_ components can improve the SO_2_ tolerance of Mn-based catalysts, because TiO_2_ can restrain the SO_2_ adsorption due to the weak interaction between SO_2_ and TiO_2_, and CeO_2_ can act as a sacrificial site to alleviate the sulfation of active MnO_x_ [1,24,27,55]. However, the reported SO_2_ resistant performance of MnO_x_–CeO_2_–TiO_2_ catalysts was limited to some extent, as listed in Table 4. The excellent SO_2_ tolerance of the MCTO-600 catalyst in this work can be attributed to its largest mesopore size with an average pore size of 32 nm compared with that of the other reported catalysts (6.7–17.3 nm), because the decomposition of ammonium sulfate occurs more easily in larger pores [29,30,31]. In addition, the above in-situ DRIFTS results showed that the NH_3_–SCR reaction over the MCTO-600 catalyst followed both the E–R and L–H mechanisms, especially for the E–R pathway; namely, the gaseous NO could react directly with the active NH_3_ species adsorbed on Lewis acid sites, which is also beneficial to the superior SO_2_ resistance ability [25,56]. 

Meanwhile, the H_2_O resistance of the MCTO-600 catalyst was also investigated, as shown in Figure 12b. When 5 vol. % of H_2_O vapor was introduced into the system, the NO conversion decreased from 100% to 90% in the first 2 h and remained stable for the next 4 h, which returned to 100% after the H_2_O vapor was cut off, which may have been due to the fact that the adsorption of H_2_O at the active sites was reversible, and the occupied active sites could be easily regenerated after H_2_O desorption [55,57]. When 100 ppm of SO_2_ and 5 vol. % of H_2_O vapor were injected into the flue gas simultaneously, as shown in Figure 12c, the NO conversion of the MCTO-600 catalyst reduced from 100% to 75% within 2 h and remained stable for the next 4 h, which then gradually increased from 75% to 95% within 2.5 h after SO_2_ + H_2_O was cut off, suggesting that the passivation of the catalyst by SO_2_ + H_2_O included reversible and irreversible parts [58]. These results indicated that the coexistence of SO_2_ and H_2_O vapor at a low temperature had a greater impact on the NO removal than that of SO_2_ alone, which may have been due to the fact that H_2_O can accelerate the deposition of sulfates on the surface of catalyst, thereby intensifying the poisoning of SO_2_ [59,60]. In addition, the stability and longevity of the catalyst are also important for practical applications, as shown in Figure 12d; the NO removal efficiency of the MCTO-600 catalyst was stabilized at 100% within 24 h under the test conditions, demonstrating its excellent long-term stability. 

## 4. Conclusions

In this work, the Mn–Ce–Ti–O composite aerogel with a large mesopore size was successfully prepared via a one-pot sol-gel method by using propylene oxide as a network gel inducer and ethyl acetoacetate as a complexing agent, and which was used for NH_3_–SCR of simulated flue gas. The results indicate that the Mn–Ce–Ti–O catalyst calcined at 600 °C exhibits the highest NH_3_–SCR activity, good N_2_ selectivity, lowest E_a_, excellent SO_2_ resistance, and long-term stability. Its highest NH_3_–SCR activity is related to the most abundant Lewis acid sites and the best reducibility among the four catalysts calcinated at different temperatures (400, 500, 600, and 700 °C). The Lewis acid sites are mainly contributed by Ce^3+^ while the Brønsted acid sites mainly by Mn^4+^. The Lewis acid sites play a more important role than the Brønsted acid sites do in our catalysts investigated. Its superior SO_2_ resistance to the other reported MnO_x_–CeO_2_–TiO_2_ catalysts can be mainly attributed to its large mesopore size with an average pore size of 32 nm. The abundant Lewis acid sites are another factor that alleviates sulfation of NH_3_, which may reduce vapor pressure of NH_3_ greatly. Both the Langmuir–Hinshelwood (L–H) mechanism and the Eley–Rideal (E–R) mechanism are involved in the NH_3_–SCR. The results of this study may provide a good opportunity for preparing and designing NH_3_–SCR catalysts with superior sulfur tolerance and low temperature activity.

## Figures and Tables

**Table 1:** The mean crystallite sizes of metal oxides of different catalysts.

Sample	D_TiO_2__ (nm)	D_CeO_2__ (nm)	D_Mn_2_O_3__ (nm)	D_Mn_3_O_4__ (nm)
MCTO-0	7.8	-	-	-
MCTO-400	9.4	-	6.8	15.3
MCTO-500	11.5	-	12.5	20.8
MCTO-600	16.2	8.0	13.0	21.5
MCTO-700	21.5	13.6	17.8	38.8

**Table 2:** Brunauer–Emmett–Teller (BET) specific surface area and pore textures of the samples.

Catalyst	Surface Area (m^2^·g^−1^)	Mean Pore Diameter (nm)	Pore Volume (cm^3^·g^−1^)
MCTO-400	127.1	14	0.44
MCTO-500	71.6	22	0.38
MCTO-600	35.8	32	0.29
MCTO-700	18.9	20	0.095

**Table 3:** Surface atomic compositions of catalysts measured by XPS.

Sample	Atomic Composition (%)					O_α_/(O_α_ + O_β_) (%)	Ce^3+^/Ce (%)	Mn^4+^/Mn (%)	Mn^3+^/Mn (%)	Mn^2+^/Mn (%)
	Mn	Ce	Ti	C	O					
MCTO-400	5.4	1.8	16.4	13.3	63.2	57.2	17.5	25.9	31.3	42.8
MCTO-500	5.5	1.3	23.1	6.6	63.5	34.9	17.9	27.9	30.6	41.5
MCTO-600	4.6	1.7	25.3	5.1	63.2	41.3	24.0	29.3	29.9	40.8
MCTO-700	4.9	2.4	24.9	2.3	65.6	29.0	13.8	24.3	30.5	45.3

**Table 4:** A comparison of anti-SO_2_ performance of MnO_x_–CeO_2_–TiO_2_ catalysts prepared by different methods in the reported literatures.

Catalysts	Average Pore Size	Preparation Methods	Reaction Conditions	NO Conversion Before and After Introducing SO_2_	Refs
MnO_x_–CeO_2_@TiO_2_	13.3 nm	three-step method	T = 180 °C, [NO] = [NH_3_] = 500 ppm, [O_2_] = 5%, [SO_2_] = 200 ppm, GHSV = 24,000 h^−1^	decline from 100% to 70%	[26]
Mn–Ce/TiO_2_	9.0 nm	co-precipitation method	T = 120 °C, [NO] = [NH_3_] = 600 ppm, [O_2_] = 3%, [SO_2_] = 700 ppm, GHSV = 40,000 h^−1^	decline from 92.5% to 34.6%	[33]
Mn–Ce/TiO_2_	6.7 nm	sol–gel method	T = 150 °C, [NO] = [NH_3_] = 800 ppm, [O_2_] = 3%, [SO_2_] = 100 ppm, GHSV = 40,000 h^−1^	decline from 100% to 60%	[24]
MnO_x_–CeO_2_/TiO_2_	13.7 nm	one-step hydrothermal method	T = 180 °C, [NO] = [NH_3_] = 500 ppm, [O_2_] = 5%, [SO_2_] = 200 ppm, GHSV = 24,000 h^−1^	decline from 100% to 47%	[26]
MnO_x_/CeO_2_–TiO_2_	17.3 nm	wet impregnation	T = 180 °C, [NO] = 200 ppm, [NH_3_] = 220 ppm, [O_2_] = 8%, [SO_2_] = 100 ppm, GHSV = 60,000 h^−1^	decline from 84% to 62%	[32]
MnCe/TNTs	13.6 nm	hydrothermal method	T = 150 °C, [NO] = 720 ppm, [NH_3_] = 800 ppm, [O_2_] = 3%, [SO_2_] = 100 ppm, GHSV = 100,000 h^−1^	decline from 92% to 84%	[12]
Mn–Ce–Ti–O composite aerogels	32.5 nm	one-pot sol–gel method	T = 200 °C, [NO] = [NH_3_] = 500 ppm, [O_2_] = 5%, [SO_2_] = 100 ppm, GHSV = 14,400 h^−1^	maintaining at 100%	in this work
